# Factors Associated With Electronic Health Record Usage Among Primary Care Physicians After Hours: Retrospective Cohort Study

**DOI:** 10.2196/13779

**Published:** 2019-09-30

**Authors:** Selasi Attipoe, Yungui Huang, Sharon Schweikhart, Steve Rust, Jeffrey Hoffman, Simon Lin

**Affiliations:** 1 College of Public Health The Ohio State University Columbus, OH United States; 2 The Research Institute Nationwide Children's Hospital Columbus, OH United States; 3 College of Medicine The Ohio State University Columbus, OH United States

**Keywords:** electronic health records, health information technology, primary care physicians

## Abstract

**Background:**

There is limited published data on variation in physician usage of electronic health records (EHRs), particularly after hours. Research in this area could provide insight into the effects of EHR-related workload on physicians.

**Objective:**

This study sought to examine factors associated with after-hours EHR usage among primary care physicians.

**Methods:**

Electronic health records usage information was collected from primary care pediatricians in a large United States hospital. Inclusion criteria consisted solely of being a primary care physician who started employment with the hospital before the study period, so all eligible primary care physicians were included without sampling. Mixed effects statistical modeling was used to investigate the effects of age, gender, workload, normal-hour usage, week to week variation, and provider-to-provider variation on the after-hour usage of EHRs.

**Results:**

There were a total of 3498 weekly records obtained on 50 physicians, of whom 22% were male and 78% were female. Overall, more EHR usage during normal work hours was associated with decreased usage after hours. The more work relative value units generated by physicians, the more time they spent interacting with EHRs after hours (β=.04, *P*<.001) and overall (ie, during normal hours and after hours) (β=.24, *P*<.001). Gender was associated with total usage time, with females spending more time than males (*P*=.03). However, this association was not observed with after-hours EHR usage. provider-to-provider variation was the largest and most dominant source of variation in after-hour EHR usage, which accounted for 52% of variance of total EHR usage.

**Conclusion:**

The present study found that there is a considerable amount of variability in EHR use among primary care physicians, which suggested that many factors influence after-hours EHR usage by physicians. However, provider-to-provider variation was the largest and most dominant source of variation in after-hours EHR usage. While the results are intuitive, future studies should consider the effect of EHR use variations on workload efficiency.

## Introduction

While some studies have suggested that electronic health records (EHRs) increase efficiency and productivity, the scientific evidence has been mixed. On workload efficiency, one study found that primary care physicians (PCPs) who used EHRs spent an extra 1.3 facetime minutes per visit and had increased their patient volume per week [1]. However, in another study, EHR implementation was found to be associated with a negative impact on productivity and efficiency in a pediatric ophthalmology clinic [2]. Moreover, systematic reviews did not demonstrate any superior productivity from the use of EHRs [3,4].

Since the introduction of EHRs, physicians have been reported to work longer hours, with many completing their data entry after clinic and at home, during evenings or weekends [5]. Sinsky et al attempted to quantify “work after work” using a diary among 21 physicians [6]. Solving the scale-up and accuracy challenge of diary-based studies, Arndt et al developed a passive observation method using the access log automatically collected by EHRs so that all PCPs at an EHR site could be studied. However, little is known quantitatively about the factors associated with the usage of EHRs after-hours [7]. Identifying these factors will provide knowledge to support recommendations for interventions that could improve the user experience, enhance efficiency, and mitigate burnout. Accordingly, this retrospective study examines variations in EHR usage of PCPs, with an emphasis on after-hours EHR use.

## Methods

### Setting

Nationwide Children's Hospital (NCH) is a large, free-standing US children's hospital that has used the Epic EHR system (Epic Systems Corporation, Verona) as its enterprise-wide system since 2006. All pediatricians who generated work relative value units (wRVUs) related to ambulatory primary care activity from January 1, 2015 to June 30, 2016 were included in this study. Work RVUs are a measure of billable service volume and complexity. Clinical data, billing information, and EHR usage data were extracted from the EHRs into a database for analysis. This study was approved by the NCH’s Institutional Review Board.

### Data

Data retrieved included physician demographics (age and gender), duration of employment (tenure), medical specialty, wRVUs generated during the study period, full-time equivalent (FTE) status, and EHR access logs. The EHR access log captures clinicians’ direct interactions with the EHR system, such as login, logout, and documentation in a patient’s chart.

We implemented the algorithm employed by Arndt et al to estimate the duration of EHR usage (ie, duration of a physician’s EHR activity) from EHR access logs [7]. This method included estimating time spent on particular activities using the EHR system’s automated event logging feature. This algorithm was validated using a time and motion study (ie, direct observation of 14 nonresident family medicine physicians by a trained medical student) [7].

EHR usage was divided into two separate time segments: normal (weekdays from 7am-6pm, using NCH workstations) and after-hours (weekdays from 6pm-7am, anytime on weekends, or anytime not using NCH workstations). All daily EHR activity durations for each physician were classified as belonging to one of these two different time segments (based on the activity timestamps) and summed for each time segment.

The main outcome variable was the duration of after-hours EHR usage. EHR access time during normal hours, along with physician age, gender, tenure, and wRVUs, were the main explanatory variables. Records for the analysis are organized into one record per physician per week, per the two time segments chosen.

### Statistical Analysis

Mixed effect linear regression models were used to analyze data, where physician age, gender, wRVUs, and EHR usage were treated as fixed effects, and both provider-to-provider variation and week to week variation were treated as random effects. Linear mixed models were used in this analysis to account for repeated measures within the same individuals. Distributions for dependent variables were analyzed to assess the normality assumption and to determine whether transformation was needed. Analyses were all conducted using R (Version 3.3.0, 2016) and the R lme4 package.

## Results

### Descriptive Statistics

A total of 50 physicians, of whom 22% were male and 78% were female, met the inclusion criteria and their descriptive statistics are presented in Table 1. The study physicians generated a total of 3498 aggregated weekly records (ie, access logs). Physicians spent approximately 16 hours weekly interacting with the EHRs during their normal work hours, while spending about 3 hours weekly after-hours.

### Statistical Modeling

Mixed effect linear regression models were fitted for three dependent variables: normal-hours EHR usage, after-hours EHR usage, and total EHR usage. Length of hospital service was omitted as an independent variable due to multicollinearity with age. FTE status was also omitted in further analyses due to missing values. Modeling results are reported in Table 2.

**Table 1:** Descriptive Statistics (N=50). All values are presented as mean (SD).

Characteristics	Men (n=11)	Women (n=39)	Total (N=50)
Age (years)	45.53 (8.20)	42.65 (9.95)	43.23 (9.69)
Length of hospital service (years)	12.03 (9.29)	8.83 (8.47)	9.48 (8.73)
Work relative value units	70.32 (51.44)	63.41 (40.04)	64.81 (42.67)
Full-time equivalent^a^ status	0.93 (0.09)	0.79 (0.19)	0.81 (0.18)
Clinical full-time equivalent^a^ status	0.56 (0.27)	0.53 (0.22)	0.54 (0.23)
Nonclinical full-time equivalent^a^ status	0.38 (0.25)	0.25 (0.20)	0.27 (0.22)
Normal-hours electronic health record usage (hours per week)	15.54 (8.91)	16.69 (9.16)	16.46 (9.12)
After-hours electronic health record usage (hours per week)	2.24 (3.04)	2.70 (3.64)	2.61 (3.53)

^a^There were 13 missing values and so this variable was not used in further analyses.

**Table 2:** Mixed regression models.

Model and characteristics			Normal-hours EHR^a^ usage	After-hours EHR usage	Total EHR usage
**Fixed effects^b^**					
	**Normal-hours EHR usage (minutes)**		—^c^	–0.045 (0.010)	—
		*P* value	—	<.001	—
	**wRVUs^d^**		0.203 (0.002)	0.042 (0.002)	0.235 (0.002)
		*P* value	<.001	<.001	<.001
	**Age (years)**		0.073 (0.0495)	–0.059 (0.035)	0.007 (0.062)
		*P* value	.13	.08	.89
	**Gender**		2.841 (1.234)	0.665 (0.907)	3.372 (1.580)
		*P* value	.02	.45	.03
	Constant		–2.083 (2.446)	2.727 (1.720)	0.883 (3.069)
**Random effects^e^**					
	Week		0.288 (0.536)	0.046 (0.213)	0.312 (0.559)
	Provider		12.838 (3.583)	6.965 (2.639)	21.107 (4.594)
**Model fitness (R^2^), (%)**					
	Fixed effects		76.4	23.4	75.5
	Random effects		14.4	52.0	16.2
	Total		90.8	75.4	91.7

^a^EHR: electronic health record

^b^Values presented as coefficients (Standard error).

^c^Not applicable.

^d^wRVU: work relative value unit

^e^Values presented as Variance (Standard error).

Analyses showed a positive relationship with statistical significance between wRVUs and normal-hours EHR usage; the more wRVUs the physician generated, the more demand for normal-hours EHR usage. There was also a statistically significant relationship between gender and normal-hours EHR usage, with females spending more time using the EHRs during normal hours than males. No associations were found with age. Provider-to-provider variability (SD 3.58) contributed substantially more to the variation of normal-hours EHR usage than the variability across weeks (SD 0.53).

For after-hours EHR usage*,* we included normal-hours EHR usage as an explanatory variable. Modeling results show a statistically significant inverse relationship between normal hours and after-hours EHR usage; the more physicians worked with the EHRs during normal hours, the fewer hours they spent after hours with the EHRs. In addition, there was another positive relationship with statistical significance found between wRVUs and after-hours EHR usage. The gender effect, while not statistically significant, appears to be as strong for after-hours use as it is for normal-hours use. Again, provider-to-provider variability (SD 2.64) contributed more to the variation of after-hours EHR usage than the variability across weeks (SD 0.21).

Total EHR usage was also studied using the same approach. The association with wRVU remained statistically significant and in the same direction as for the normal-hours model. Similarly, the model suggested an association between gender and total EHR usage, with female physicians spending more time than males.

## Discussion

### Principal Results

In this study, physicians on average spent about 16 hours weekly interacting with the EHR during their normal work hours, while spending about 3 hours weekly after hours. Thus, assuming a 2.5-day clinical work week (based on the average cumulative FTE of our sample group), our findings suggest that physicians may be spending about 6.4 hours during normal work hours and 1.2 hours after work, per weekday, completing EHR tasks. These findings are somewhat comparable to those of Arndt et al, as PCPs in their study spent 4.5 hours and 1.4 hours each weekday completing EHR tasks during and after hours, respectively [7]. Previous studies have reported time spent completing EHR tasks ranging from 2.4 to 5.9 hours per weekday; however, few of these studies specified if this time was during or after work hours [7-11].

The current study also found a positive association between wRVUs and EHR usage. This finding is expected, as wRVUs reflect the volume and intensity of medical services provided, thus the higher the wRVUs, the more likely a physician is to spend time with the EHRs. Work RVUs are one measure of physician productivity and have been found by other researchers to have a positive relationship with EHR usage [12,13]. Moreover, gender was significantly associated with normal and total EHR usage, with women spending more time with the EHRs. This association was not seen with after-hours EHR use, but perhaps while the association is the same, there is an absence of statistical significance. Nonetheless, gender differences in the use of EHRs, both during normal hours and after hours, have not been clearly documented in the scientific literature. Further, age was not found to be associated with normal or after-hours EHR use, which is somewhat consistent with findings in the scientific literature [1,14].

An unexpected finding from this study is that the provider-to-provider variation of after-hours EHR usage time was far larger than any of the other factors we examined. In terms of the variation of after-hours time spent with EHRs every week, the provider-to-provider variation explained half (52%) of the variance, whereas all fixed effects combined (wRVU, age, gender, normal-hours EHR usage) only explained 23% of the variation. Overall, the model effectively explains 75% of all the variations seen. Accordingly, one potential approach to reduce after hours use is training, which could potentially reduce the variation between providers. To our knowledge, this is the first study to quantify the effect size of this variation among providers on EHR usage.

### Limitations

There are a few limitations of this study. First, the study sample size is small and limited to a single practice, a single type of provider, and a single commercial EHR system, thus limiting the generalizability of study findings. We focused our study on PCPs only, because the on-duty versus off-duty hours of hospitalists and other specialties are technically more complicated to study. Second, even though we chose to limit our study to PCPs, the use of clock time to define work hours without factoring in individual physician schedules is suboptimal. Tailoring after-hours usage to each physician’s schedule would be ideal. Third, although validated by Arndt et al, the usage hours estimated from the access logs may only be a proxy of the actual hours spent using the EHRs. Fortunately, if there is an estimation bias, it equally affects the normal-hours and after-hours usage. Fourth, some EHR activities are not patient care specific. It would be interesting to assess the differential effects of patient care–specific and nonpatient care–specific EHR activities. In addition, we did not have complete FTE status information on our sample, which further weakens the generalizability of the study findings and conclusions that can be drawn. Further, the reasons for the current findings were not empirically established. Future studies, using a mixed methods approach could shed light on reasons for these observations.

### Conclusions

The present study filled a gap in the literature to statistically model variations in the duration of EHR use among PCPs and identified some of the factors that influence after-hours EHR use. These findings are essential to empirically establish these associations and advance our knowledge on this topic.

## Abbreviations

EHR: electronic health record
FTE: full-time equivalent
NCH: Nationwide Children's Hospital
PCP: primary care physician
wRVU: work relative value unit
